# Translational Science: Epistemology and the Investigative Process

**DOI:** 10.2174/138920209787847005

**Published:** 2009-04

**Authors:** Edward R Dougherty

**Affiliations:** Department of Electrical and Computer Engineering, Texas A&M University, Computational Biology Division, Translational Genomics Research Institute, USA

## Abstract

The term “translational science” has recently become very popular with its usage appearing to be almost exclusively related to medicine, in particular, the “translation” of biological knowledge into medical practice. Taking the perspective that translational science is somehow different than science and that sound science is grounded in an epistemology developed over millennia, it seems imperative that the meaning of translational science be carefully examined, especially how the scientific epistemology manifests itself in translational science. This paper examines epistemological issues relating mainly to modeling in translational science, with a focus on optimal operator synthesis. It goes on to discuss the implications of epistemology on the nature of collaborations conducive to the translational investigative process. The philosophical concepts are illustrated by considering intervention in gene regulatory networks.

## INTRODUCTION

Google reports more than 100,000 results for "translational science." These include a host of institutes, centers, and programs, dealing exclusively with medicine – at least at the top of the hit list – although the phrase “translational science” itself does not imply any specific discipline. For the most part the phrase is used as if everyone somehow knows what it means, so that no definition is required. Given the fact that the term “science” is used everywhere to refer to activities and theories well outside the proper epistemological domain of science, one can hardly expect a phrase based on the modification of the term “science” to possess a universal implicit meaning.

James Levine summarizes the situation when he writes,

What is translational research? Can I start dreaming of my children becoming translational researchers? The wonderful thing about translational research is that every one knows exactly what it means – the only trouble is that none of them have the same definition. One senior colleague told me that translational research ‘is to bring the gene from the laboratory to the bedside,’ whereas another colleague’s definition was ‘shifting science to the community – outreach they call it’ [1].

This is not to say that efforts have not been made. For instance, Donna Johnstone makes a good attempt at providing a definition commensurate with a general view in the world of drug discovery:

What is translational science? This is a question I get asked frequently, by both academic and industrial scientists trying to understand the ‘new kid on the block’ in terms of placement within a bioscience discipline. Rather than go through the well worked phrases of science that goes ‘from bench to bedside’ or from ‘mouse to man’, a more accurate definition might be ‘the application of biomedical research (pre-clinical and clinical), conducted to support drug development, which aids in the identification of the appropriate patient for treatment (patient selection), the correct dose and schedule to be tested in the clinic (dosing regimen) and the best disease in which to test a potential agent (disease segment)’ [2].

While Johnstone succeeds in bringing focus on the application, she does not address the scientific meaning of “translational science”. The goal is stated but not the implications of the enterprise being scientific.

The goal of translational science is clear: connect scientific knowledge and the application of that knowledge. If translational science is be a meaningful endeavor, then the kind of knowledge represented by the connection must be understood. Moreover, to the extent that translational science refers to genomics, proteomics, and high-dimensional biology in general, the ubiquitous epistemological shortcomings in these areas [3, 4] surely imply that it should receive careful epistemological scrutiny.

This paper discusses epistemological issues relating to translational science and the manner in which epistemology affects the investigative process. The focus is on translational modeling, how it is a natural extension of scientific modeling with the added ingredient of purposeful action in the physical world, and how the demands of modeling shape translational research. The philosophical concepts are illustrated by considering intervention in gene regulatory networks.

## MODERN SCIENCE

Because the epistemology of translational science grows out of the scientific epistemology, of necessity we must begin with the latter, albeit, briefly but hopefully sufficient to make the overall exposition self-contained (referring to [4, 5] for those interested in an in-depth discussion relative to genomics and, more generally, to computational biology).

The great transformation of science, from the science of antiquity and the medieval period to the beginning of modern science, occurred in the Sixteenth Century when Galileo recognized the need for designed experiments – methodological as opposed to unplanned observation. Observations would be made under experimental constraint to elicit specific responses that would be integrated into a conceptual system. In *The Rise of Scientific Philosophy*, Hans Reichenbach writes, “As long as we depend on the observation of occurrences not involving our assistance, the observable happenings are usually the product of so many factors that we cannot determine the contributions of each individual factor to the total result” [6]. Perhaps Immanuel Kant coined the perfect metaphor for pre-Galilean science when, in the preface to the second edition of the *Critique of Pure Reason*, he wrote, “Reason must approach Nature… [as] a judge who compels witnesses to reply to those questions which he himself thinks fit to propose. To this single idea must the revolution be ascribed, by which, after *groping in the dark* for so many centuries, natural science was at length conducted into the path of certain progress” [7].

Knowledge can be obtained by groping in the dark, but groping lacks efficiency and what morsels are found are unlikely to readily fit into a conceptual system. As Kant says, Nature should be probed with questions that the scientist “thinks fit to propose”. Arturo Rosenblueth and Norbert Wiener state, “An experiment is a question. A precise answer is seldom obtained if the question is not precise; indeed, foolish answers – i.e., inconsistent, discrepant or irrelevant experimental results – are usually indicative of a foolish question” [8]. It is true that a scientific question might arise from the chance observation of some phenomenon that is inconsistent with existing theory, but once that inconsistency is observed, it is the scientist’s appreciation of the inconsistency that leads to precise questions that must be addressed by experiments designed to elicit answers that lead to a reformulation of the theory in such a way as to rectify the inconsistency. But inconsistency is only one motivation for new experiments; another, and probably more prevalent one, is incompleteness. Here the scientist recognizes the existence of phenomena not accounted for by existing theory, speculates on how the theory might be expanded to include these phenomena, and designs experiments targeted to achieve that expansion. This modern, post-Galilean approach to science is today often rejected in favor of a pre-Galilean groping in the dark, albeit, one enhanced by very fast high-performance groping called “data mining”, where conceptually driven experimentation is abandoned. Just increase the amount of data and some pattern will turn up! Indeed, clustering algorithms form clusters. Classification rules form classifiers. But do the resulting clusters and classifiers embody scientific knowledge? [9] Post-Galilean experimental design is to pre-Galilean groping as planned agriculture is to hunting and gathering.

Experiments drive the epistemology of science and the product of an experiment is a set of measurements. These form the empirical basis for knowledge. In themselves, measurements do not constitute scientific knowledge. Scientific knowledge is constituted *via *systematic organization of the observed measurements, which are related to variables and relations among the variables. A system of variables and their relations forms a mathematical model. The model must be mathematical because it relates measurements *via *numerical structures or judgments *via *logical constructs. A basic model may be formed by some set of relations, say a stochastic model of a gene regulatory network, but knowledge does not stop there. Mathematical deduction leads to the full knowledge inherent in the relations. In Kantian terminology, the mathematical model *constitutes* the object of our knowledge. The experiment and the mathematical model form two inseparable requirements for scientific knowledge. Either without the other cannot yield scientific knowledge.

A mathematical model alone does not constitute a scientific theory. The model must be predictive. It must lead to experimental predictions in the sense that there are relations between model variables and observable phenomena such that experimental observations are in accord with the predicted values of corresponding model variables. There must be a predictive framework for validation because the scientific truth, or validity, of the model depends on the accuracy of predictions arising from the model. This requires the conceptual system to be related to the experimental methodology. Reichenbach states, “The reference to verifiability is a necessary constituent of the theory of meaning. A sentence the truth of which cannot be determined from possible observations is meaningless… The verifiability theory of meaning is an indispensable part of scientific philosophy” [6]. Verification of a system requires that the symbols be tied to observations by some semantic rules that relate not necessarily to the general principles of the mathematical model themselves but to conclusions drawn from the principles. In other words, the theory is checked by checking measurable consequences of the theory. These *operational definitions*, as they are called, are an intrinsic part of the theory, for without them there would be no connection between the principles and observation. There must be a well-defined procedure for relating the consequences of the equations to quantifiable observations, such as gene expression in the steady state of a gene regulatory network. A scientific theory must have two parts: a structural model and a set of operational definitions for its symbols.

Since a model can only be verified to the extent that its symbols can be tied to observations in a predictive framework, limitations on the ability to design and perform experiments engender limitations on the complexity of a theory. In producing a verifiable theory, the theorist cannot exceed the experimentalist's ability to conceive and perform appropriate experiments, nor can the experimentalist produce directly meaningful experiments unless they are designed with a symbolic structure in mind. Mathematics alone is divorced from the empirical basis of science, but without mathematics, meaningful experiments are impossible because scientific meaning is ultimately determined by a set of relationships between a mathematical system and experimental measurements. Indeed, because a model consists of mathematical relations and system variables must be checked against quantitative experimental observations, there is no nonmathematical way to describe the requirements and protocols to assess model validity.

## TRANSLATIONAL SCIENCE

In discussing the meaning of translational science, we can turn to a definition in Webster’s Dictionary: to translate is “to change into another medium or form; as, *translate* ideas into action” [10]. Translational science transforms a scientific mathematical model, whose purpose is to provide a predictive conceptualization of some portion of the physical world, into a model characterizing human intervention (action) in the physical world. Whereas the pure scientist typically tries to minimize human interference, translational science extends science to include conceptualization of human-originated action in the physical world and thereby raises epistemological issues relating to the knowledge of this intentional intervention into the natural order. Scientific knowledge is translated into practical knowledge by expanding a scientific system to include inputs that can be adjusted to affect the behavior of the system and outputs that can be used to monitor the effect of the external inputs and feed back information on how to adjust the inputs.

One must be careful in trying to make a crisp division between science and translational science. Scientific experimentation is not a passive enterprise: Nature is probed according to the plan of the scientist. The scientific enterprise is pragmatic in that its concept of truth depends on predictions in the future, so that scientific knowledge is contingent, always open to refutation by new observations. Reichenbach writes, “Scientific philosophy has constructed a *functional* conception of knowledge, which regards knowledge as an instrument of prediction and for which sense observation is the only admissible criterion of nonempty truth” [6]. Translational science simply goes a step further. Its purpose is to characterize intentional intervention in the physical world for the purpose of attaining a desired end. Moreover, mathematical models constitute translational scientific knowledge as well as scientific knowledge. Since any physical action upon a physical system brought about by human action must be understood in terms of measurements relating to those physical actions, such as the amount of electrical charge, the translational scientific model is itself a scientific model. In a sense, it is the purpose to which the model is put that determines its translational character. Arturo Rosenblueth and Norbert Wiener go so far as to make a universal claim of intention, and therefore a unification of science and translational science, when they write, “The intention and the result of a scientific inquiry is to obtain an understanding and a control of some part of the universe" [8]. For them, science and translational science are inextricably linked, the ultimate purpose of acquiring scientific knowledge being to translate that knowledge into action. The question is how that translation is to be accomplished.

If one is going to transform a physical process, then the conceptualization of that physical transformation takes the form of a mathematical operator on some mathematical system, which itself is a scientific model for the state of nature absent the transformation. There are two basic operator problems concerning systems. The first is *analysis*: given a system, *S*, and an operator, **ψ**, what can we say about the properties of the output system, **ψ**(*S*), in terms of the properties of *S*? It might be mathematically difficult to characterize completely the output system given the complete input system or we may only know certain properties of the input system, so that the best we can hope for is to characterize related properties of the output system.

For illustration purposes, consider a gene regulatory network (GRN) operating in a constant environment. Here we assume that a GRN consists of a finite number of genes with various regulatory relations among them such that two conditions are satisfied: (1) the state of the network, which consists of the vector of all gene values, at time *t* depends only on gene values at time *t* – 1, so that the state vector is dynamically described by a Markov chain; and (2) the Markov chain possesses a steady-state distribution characterizing the long-run dynamics of the network. If **ψ** is some structural operation on the GRN conceptualizing an action upon the structure (wiring) of the physical network, then a key problem for analysis is to characterize the steady-state distribution of the output network, **ψ**(*S*), in terms of the mathematical representation of **ψ** and the steady-state distribution of the input network, *S* [11]. From a medical perspective, **ψ** might correspond to the permanent blocking of a gene product and the pragmatic translational issue might be to quantify the effect on the long-run survivability of the patient with intervention as opposed to without intervention.

The second basic operator problem is *synthesis*: given a system, we would like to design an operator to transform the system in some desirable manner. Unlike analysis, in synthesis it is the design of the operator that is important. Whereas the purpose of science, absent translation, is to gain knowledge of the natural world, translational science is about changing it, and synthesis is the act of designing operations to make those changes. It represents the critical act for human intervention and forms the existential basis of engineering. One could proceed in a trial-and-error manner, trying one operation after another and observing the result. In this case the operator is not constructed based on knowledge of the scientific system and synthesis is not part of translational science; rather, it is a form of groping in the dark, where one tries one operation after another in the hope of getting lucky, operator mining instead of data mining. Such groping in the dark does not preclude analysis, and therefore does not preclude translational scientific knowledge; however, the critical engineering aspect, that being operator creation for the purpose of transforming nature, is not translational in the scientific sense.

For synthesis to properly occur within translational science requires that synthesis begin with a mathematical theory constituting the relevant scientific knowledge and the theory be utilized to arrive at an optimal (or close to optimal) operator for accomplishing the desired transformation under the constraints imposed by the circumstances. The classic example, the one commencing the era of modern engineering, involves optimal time series filtering in the classic work of Andrey Kolmogorov [12] and Norbert Wiener [13] (Although [13] was published in 1949, an unpublished version appeared in 1942). One begins with a scientific model and expands the model by adjoining operators with which to desirably alter the behavior of the original system. A criterion exists by which to judge the goodness of the response and the goal is to find an optimal way of manipulating the system. In the classic Wiener-Kolmogorov theory, the scientific model is a signal and the translational problem is to linearly operate on the signal so as to transform it to be more like some ideal (desired) signal. The synthesis problem is to find an optimal weighting function and the goodness criterion is the mean-square difference between the ideal and filtered signals.

Andrey Kolmogorov is generally considered the premier probabilist of the Twentieth Century; Norbert Wiener is generally considered the father of modern engineering – and here we mean engineering in the sense of translational science, its more modern role, not in the sense of building devices, its more historical role. The Romans were great engineers but they were not translational scientists. Modern engineering is mathematical engineering, applied mathematics with a driving translational purpose. Whereas the pure mathematician is motivated by internal mathematical questions, the applied mathematician develops mathematics for science or engineering. Both can be excellent mathematicians; it is just that their domains are different – although there is certainly no clear line of demarcation between them. The theoretical physicist and theoretical biologist are of necessity practitioners of applied mathematics. Their expertise is quite different from the experimental physicist or experimental biologist, who must design experiments to answer questions or validate hypotheses arising from theoretical speculation. All of these categories lie on a continuum; nevertheless, they must be recognized because each contributes to scientific knowledge in its own way. Here, when the term “engineer” is used, it will refer to the modern mathematical engineer, our interest being in synthesis within translational science.

Synthesis *via *mathematical optimization within the framework of a scientific model does not mean that one can obtain a corresponding physical transformation, but it does provide both a target for physical design and a benchmark for performance. Vladimir Pugachev writes,

The theory of optimal operators does not enable operators to be found directly which can be embodied forthwith in real constructions. It only enables those mathematical operations on input signals to be determined for which the theoretical limit of accuracy is achieved, for given probability characteristics of the mode of operation and noise, having regard to the nature of the problem and the intrinsic properties of the available data. Accordingly, the practical value of the theory of optimal operators consists mainly in the fact that it makes possible the determination of the theoretical optimum towards which the design engineer must strive in designing a real control system [14].

Having conceptualized the translational problem and found an optimal solution within the mathematical formalization of the problem, the scientist and mathematical engineer can now turn to the technological design engineer to build a device that acts in the physical world in a manner corresponding to the optimal operator within the translational scientific model – or at least approximates to a satisfactory degree the action of the optimal operator.

## INTERVENTION IN GENE REGULATORY NETWORKS 

To illustrate synthesis in translational science, we consider therapeutic intervention over time in a gene regulatory network. Given a GRN model, the translational problem is to arrive at a series of intervention decisions whose objective is to decrease the long-run likelihood of states favorable to pathological cell functionality. To accomplish this goal, the task of finding an effective intervention strategy has been formulated as a classical sequential decision making optimization [15]. Two kinds of costs contribute to measure the goodness of an intervention at any stage in the treatment process: (1) a cost that discriminates between the desirable and undesirable states of the system, and (2) a cost of intervention that quantifies the negative effect of an intervention, say, drug treatment or chemotherapy. These are combined into a total cost per stage and the objective of the decision maker is to minimize the accumulated cost associated with the progression of the network. To wit, given the state of the network, an effective intervention strategy identifies which action to take so as to minimize the overall cost. The devised intervention strategy can be used as a therapeutic strategy that alters the dynamics of aberrant cells to reduce the long-run likelihood of undesirable states favorable to the disease.

In this framework, the translational problem assumes the existence of an external regulator and a binary intervention input *u*(*t*) at each stage *t*. The value, 0 or 1, of the intervention input *u*(*t*) specifies the action on a control gene. Treatment alters the status of the control gene. If treatment is applied, *u*(*t*) = 1, then the state of the control gene is toggled; otherwise, the state of the control gene remains unchanged. Given the cost-per-stage function, the objective is to derive an optimal intervention strategy from among a class of allowable strategies. Essentially, a strategy is a function that, at each stage, takes as input the current state (and perhaps the history) of the GRN and outputs a decision: *u*(*t*) = 0 or *u*(*t*) = 1. The decision maker searches for an optimal strategy that minimizes the expected cost aggregated over the long-run progression of the GRN. These kinds of problems have a long history in control engineering [16, 17].

We illustrate the kind of results one can obtain with an optimal GRN intervention strategy based on external control, where in this case the GRN is a probabilistic Boolean network [18]. The intervention objective is based on a study in which experimentally increasing the levels of the Wnt5a protein secreted by a melanoma cell line *via *genetic engineering methods directly altered the metastatic competence of that cell as measured by the standard *in vitro* assays for metastasis and in which an intervention that blocked the Wnt5a protein from activating its receptor, the use of an antibody that binds the Wnt5a protein, substantially reduced Wnt5a's ability to induce a metastatic phenotype [19]. These observations suggest a control strategy that reduces the WNT5A gene's action in affecting biological regulation, because disruption of this influence could reduce the chance of a melanoma metastasizing, a desirable outcome. In [20], a 7-gene network containing the genes WNT5A, pirin, S100P, RET1, MART1, HADHB and STC2 was considered. Desirable states were those in which WNT5A was down regulated, the control gene was pirin, a cost function was defined to reflect the goal and the cost of intervention, and the optimal long-run control strategy was derived by dynamic programming optimization techniques. Fig. (**1a**) shows the original steady-state distribution of the GRN absent control and Fig. (**1b**) shows the steady-state distribution of the controlled network. We observe a marked decline in the undesirable probability mass in the controlled network.

The classical intervention optimization just described has two drawbacks. First, it requires full knowledge of the Markov chain associated with the GRN and, second, the computational complexity of the optimization algorithm increases exponentially with the number of genes in the model. From the estimation perspective, one should note that the control strategy has not been derived from the GRN model itself, but rather from the Markov chain derived from the model. Owing to the amount of data required, it is difficult to estimate the transition probability matrix determining the Markov chain, but it can be much more difficult to estimate the full GRN model. The Markov chain is a *characteristic* of the GRN that provides a partial description. Another characteristic is the regulatory (connectivity) graph that shows the existence of regulatory relations among the genes but not the relations themselves. Both the estimation and computational impediments to the design of control strategies can be eased by utilizing only partial information regarding the model. The resulting policy is typically suboptimal from the perspective of the full model, but it may be all that is possible given the estimation and computational requirements of the full model.

One long-run policy, based on the mean first passage times of the network, is based on two motivations: (1) it is preferable to reach desirable states as early as possible; (2) it is preferable to leave undesirable states as early as possible [21]. Given a control gene, if without intervention a desirable state reaches the set of undesirable states on average faster than with intervention, then the decision is to intervene and force the trajectory of the model to continue from the new state resulting from flipping the value of the control gene. If with intervention an undesirable state reaches the set of desirable states on average faster than without intervention, then again the decision is to intervene. These insights motivate the use of mean first passage times for designing intervention strategies. Given time-course measurements, it is not necessary to estimate the full GRN or even the associated Markov chain; one need only estimate the mean first passage times from each desirable state to the set of undesirable states and, vice versa, from each undesirable state to the set of desirable states. These mean first passage times constitute the required network characteristics for the algorithm and only they are estimated, thereby greatly reducing the estimation problem. In addition, the computational problem is mitigated because one need not use a complex dynamic programming algorithm to solve the full optimization relative to a cost function.

If we simply focus on the difficulty of inferring a full model, then there are two classic engineering approaches that can be employed; adaptive control [22] and robust control [23]. In adaptive control, the model and the control strategy are simultaneously estimated online as the data sequence is input into the adaptive algorithm. Not only does this avoid the need for full model estimation, it also allows the controller to adapt to changes in the underlying physical processes that would perturb the model. With robust control, it is assumed that the model, or the characteristic of the model being employed, is not known with certainty, so that the model is assumed to belong to an *uncertainty class*, and the control strategy is designed to take into account the performance across the entire uncertainty class. For instance, in the case of GRNs, robustness can be with respect to regulation or the effect of latent variables on the model [24]. Adaptive and robust methods result in control strategies that are suboptimal relative to optimization for the full model, but they often provide good performance when certain knowledge of the full model is not available. Whereas the scientist might not be satisfied with such uncertainty with regard to the scientific model, the translational scientist has an application in mind and poses the problem in a way compatible with the state of partial knowledge. If the goal is curing patients, then the experimentalist needs to be guided by the engineer to design the kind of experiments that best support the translational goal and the engineer needs to be guided by the clinician as to the kind of effects that the translational solution should provide.

## THE INVESTIGATIVE PROCESS

The epistemology of translational science tends to place certain demands on the investigative process in order that it be successful, in particular, the nature of the complementary expertise and of the interaction among researchers from different backgrounds. While these have been worked out over many decades in the traditional engineering disciplines, such as electrical and mechanical engineering, they present challenging problems for medicine. Wiener recognized the difficulties that the mathematical requirement of science and translational science would present for medicine when, in 1948, he wrote the following words:

If a physiologist who knows no mathematics works together with a mathematician who knows no physiology, the one will be unable to state his problem in terms that the other can manipulate, and the second will be unable to put the answers in any form that the first can understand. Dr. Rosenblueth has always insisted that a proper exploration of these blank spaces on the map of science could only be made by a team of scientists, each a specialist in his own field but each possessing a thoroughly sound and trained acquaintance with the fields of his neighbors; all in the habit of working together, of knowing one another's intellectual customs, and of recognizing the significance of a colleague's new suggestion before it has taken on a full formal expression. The mathematician need not have the skill to conduct a physiological experiment, but he must have the skill to understand one, to criticize one, and to suggest one. The physiologist need not be able to prove a certain mathematical theorem, but he must be able to grasp its physiological significance and tell the mathematician for what he should look [25].

Wiener’s statement provides an investigative framework for translational research. The GRN control synthesis problem previously discussed can be decomposed into several aspects: (1) construct the GRN: (2) define the optimization problem; (3) solve the optimization problem; and (4) physically implement the optimal therapy. One might argue that, unless a reliable model exists, the costs can be medically determined, and the optimal therapy implemented in treatment, then posing and solving the optimization problem is of little benefit. On the contrary, the existence of a translational mathematical system can guide the scientist in building a model that can be fruitfully applied, the clinical researcher in studying costs and benefits that accrue from certain kinds of interventions, and the biological technologist in devising methods of intervention that can lead to improved patient care. Only within a conceptual framework and in collaboration can the experimentalist, clinical researcher, and technologist ask the right questions and look to devise the right techniques. By providing the conceptual framework in the form of a mathematical model, engineering is the glue that holds all of this activity together. In a properly functioning relationship, the scientist does not hand the engineer a set of experimental data and ask the engineer to find something in it; to wit, the comment of Rosenblueth and Wiener that “foolish answers” result from imprecise questions. When there is a translational purpose, the overall enterprise must be guided by that purpose.

It is here that the purview of the (non-translational) scientist and the translational scientist diverge. The scientist may be interested in finding many relationships between many variables; for instance, the goal might be to build fine-grain networks including as many genes and proteins as possible. In this sense, there is no end to the scientist’s endeavor; discover as many relationships among as many variables as possible so as to continue to build the system to be ever more encompassing. The purpose of the translational scientist is quite different. For translation, a critical issue is to form the conceptualization at the right level of abstraction. The model must be sufficiently complex to permit the translational problem to be formulated within it to a degree sufficient for the application at hand and it must be simple enough that the translational problem is not obscured by too much structure, the necessary parameters can be well enough estimated, and the optimization is mathematically and computationally tractable. The desire for simplicity drives much of the work of engineers: reduce (compress) the model to achieve tractability while at the same time keeping sufficient information so that the resulting solution, while suboptimal from the perspective of the full model, is still acceptable. One need only look at the great effort expended on image compression to recognize the importance of model reduction. A basic way of overcoming computational limitations when designing intervention strategies on GRNs is to reduce the GRN model [26]. It is important for the success of the translational enterprise that there be tight interaction between the scientist and engineer when it comes to model complexity.

There can be many ways of formulating a mathematical model constituting the same scientific knowledge, but the *right* representation (mathematical formulation) can be crucial both for recognizing how to pose the translational problem and for solving it. Many problems have stood unsolved for some time until someone discovers the appropriate transformation of the system that puts it in a form more suitable to solution (sometimes very easily once the system is transformed). One can hardly imagine signal processing without the use of Fourier series to represent signals. In addition to facilitating a mathematical solution, the proper formulation may pave the way for successful implementation of the physical system.

Perhaps the most striking aspect of Wiener’s comment is with regard to the nature of the collaboration implied by the interaction just described. The mathematician (engineer) must be an expert mathematician and possess the ability to criticize and suggest appropriate experiments. The scientist (physiologist) must be an expert scientist and be able to grasp the scientific significance of a mathematical proposition and guide the mathematician in the process of symbolic formalization. To form such partnerships is not easy. It takes great effort and the bringing together of scientists and engineers with exceptional expertise and talent. Among the impediments to the progress of translational science in the medical domain, Arthur Feldman includes, “a paucity of well-trained multi- and inter-disciplinary investigative teams” [27]. It is important to understand Feldman’s comment in the light of Wiener’s 1948 statement, the key phrase being “investigative teams”. Neither Feldman nor Wiener is calling for multi-disciplinary individuals. These would be neither expert scientists, nor expert mathematicians. Wiener is not talking about having people with some smattering of scientific or mathematical knowledge; rather, “each is a specialist in his own field”. It does little good for a brilliant biologist to surround himself with assistants possessing only superficial training in mathematics, nor for a first-rate engineer to work with any but the best biologists. Arturo Rosenblueth was a brilliant physiologist and he chose to work with the greatest mathematical engineer of the Twentieth Century. Together they produced seminal work in systems biology [28]. While in the past it may have been possible for a single individual to bring into one mind all the science and mathematics to make transformational progress at the frontier of knowledge – Isaac Newton being the prime example – there is just too much to know in contemporary science and mathematics. For instance, the ability to obtain biological data is growing at an enormous rate and a vast set of relations between genes and proteins far outstrips the ability of any single person to grasp any but a tiny subset. Biological systems are more complex and exhibit greater nonlinearity than the humanly designed systems of classical engineering, thereby requiring fundamental mathematical knowledge for modeling these systems.

Consider three basic problems of translational genomics: classification, clustering, and the control of regulatory networks. Classification is an old, non-intuitive, and difficult subject requiring a rigorous education in multivariate probability theory. Clustering concerns random labeled point sets, a much harder subject than multivariate probability theory. The study of gene regulatory networks lies within the theory of stochastic nonlinear dynamical systems, one of the most difficult mathematical areas, and the difficulty only increases when one includes the theory of control. More generally, translational genomics, in particular, its roles in diagnosis and regulatory therapy, rests on several engineering disciplines: signal processing, communication theory, control theory, information theory, and pattern recognition. These are among the most difficult mathematical areas within engineering and, for the most part, they have historically resided within electrical engineering. None of this should be surprising. In 1948, Wiener wrote, “The group of scientists about Dr. Rosenblueth and myself had already become aware of the essential unity of the set of problems centering about communication, control, and statistical mechanics, whether in the machine or in living tissue” [25]. By 1948, Wiener had become aware of the epistemological unity of systems and translational science concerning systems, be it electrical systems and communication theory or biological systems and medical theory. There is a key difference, however, between electrical and biological systems: whereas many classical electrical engineering problems are either linear or can be approximated by linear systems, biological systems are inherently nonlinear and therefore mathematically much more difficult.

Mathematical modeling is an epistemological requirement for both science and translational science and lack of mathematical rigor represents an epistemological failure. With regard to translational genomics, one need only briefly glance at the genomic classification literature and see the ubiquity of cross-validation error estimation in situations where such an error estimate has virtually no correlation with the true error to see the practical cost of inattention to sound epistemology [29]. Beneficial application – curing patients – demands sound epistemology; otherwise, physicians cannot be assured that the translational methods provided to them are sound.

On this note, let us close with two rhetorical questions to the translational scientist. If you are suffering from diffuse large B-cell lymphoma, would you leave your treatment in the hands of a hospital technician or would you go to an expert in the treatment of lymphoma? But even prior to that, if you go to your physician for a diagnosis, would you want the proteomic classifier he or she is using to have been designed by someone who has taken one course in pattern recognition or by an expert who understands the fundamental theory and conundrums of a subject whose correct understanding often requires the overcoming of ordinary intuition?

## Figures and Tables

**Fig. (1):**
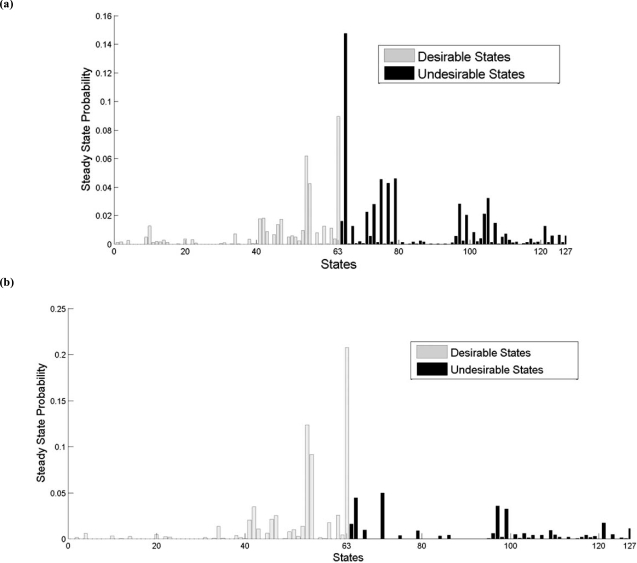
Steady-state distribution of GRN: (a) original network; (b) with intervention.
